# Books and Magazines

**Published:** 1905-05

**Authors:** 


### Books and Magazines.

OPERATIVE SURGERY. By Joseph D. Bryant, M. D., Professor of the Principles and Practice of Surgery, Operative and Clinical Surgery, University and Bellevue Hospital Medical College; Visiting Surgeon to Bellevue and St. Vincent's Hospitals; Consulting Surgeon to the Hospital for Ruptured and Crippled, Woman's Hospital, and Manhattan State Hospital for the Insane, etc. In two 8vo volumes. 1302 pages. 1576 illustrations, 90 of which are in color. Sold by subscription. Price, cloth, $10. D. Appleton & Co., Publishers, 436 Fifth avenue, New York.

This work, now appearing in its fourth edition, thoroughly rewritten, and, in fact, re-set, and printed from new plates, we believe will continue to hold its place, as it has for a number of years, as the *best work upon operative surgery.* This edition contains about 250 more pages than the previous edition and about 200 more illustrations. A large number of the older illustrations have been re-drawn. The very latest researches upon this subject have been brought out, and the reader will find in this work, operations treated and methods mentioned, which he will find in no other book or works. Special pains have been taken with the revision of "Results of Operations"; this is an important subject to the surgeon and to the general practitioner.

In the first volume the subjects of annassthesia, shock, ligatures of the innominate artery, opening of the mastoid, intracranial neurectomy, and goitre have been extensively elaborated, as have also the operations for aneurism, and that for facial paralysis, special operations on the head of the humerus, paraffin injection for correcting nasal deformity, and oesophagostomy, are some of the recent additions.

In the second volume the additions are of the widest scope. Operations on the stomach and intestines, the newer methods of intestinal anastamosis, in which there have been material advancement of late, have been brought thoroughly up-to-date. There have been added the anastamoses of Connell's suture, O'Hara's forceps, Coffey's crushable potato bobbin, Harrington's segmented rings, Lundholm's forceps, and McGraw's elastic ligature; also McGraw's recent method of colectomy, Monynihan's gastro-enterostomy, Mayo's pylorectomy, and Finney's pyloroplasty have been added. Gastropexy, gastric ulcer, epiploxyy, choledoctomy, hepatic abscess, and drainage of the bile ducts have been largely amplified. These subjects are all illustrated with new drawings, and the subjects made as interesting as possible, in Dr. Bryant's clear and concise language, and where there is any doube in the author's mind as to the real worth of an operation or what method it is clearly stated.

In the text on the kidney, the recent anatomical considerations in connection with the operations of nephrolithomy are emphasized. Nephropexy, nephrectomy, and wounds of the kidney have been added to, and the subjects of renal decapsulation introduced. Particular pains have been taken with the illustrations of tumors of the pancreas and subphrenic abscess; but here we might as well say, that particular pains have been taken with all of the illustrations that they may be absolutely correct.

On the topic of Hernia there are introduced Halsted's latest operation for radical cure of inguinal, Gordon's method for the radical cure of femoral, and Blake's and May's methods for the radical cure of umbilical. The chapter on the Anus and Rectum, and operations connected with the perineum have been revised and re-written by Prof. W. C. Lusk, of Bellevue Hospital, and among other changes are the introduction of Robson's Anoplasty, Mitchell's operation for hemorrhoids, Quenu's perineal and abdomino-perineal, proctetomy, sigmoidorectostomy and anal fissure. In the consideration of prostatectomy Watson's Proust, Alberran, and Young have been consulted. The Bottini operation for hypertrophoid prostate and Chetwood's modification have been included. On the bladder can be found Madyl's operation for extroversion and Tilden Brown's irrigating cystoscope. Becker's and others' plans of action are a part of new matter relating to the surgery of the penis.

The new things which have been mentioned above give but slight conception of the up-to-dateness of this comprehensive work. Among the miscellaneous operations are added Blake's suture for fractured pastella and also the very recent work on plastic surgery of the external ear.

A Text-Book of Medical Chemistry and Toxicology. By James W. Holland, M. D., Professor of Medical Chemistry and Toxicology, and Dean, Jefferson Medical College, Philadelphia. Octavo volume of 600 pages, fully illustrated, including eight plates in colors. Philadelphia and London: W. B. Saunders & Co., 1905. Cloth, $3, net.

Dr. Holland possesses the faculty of making even the most difficult and complicated chemical theories and farmula easy and clear. This is probably due to his thirty-five years of practical experience in teaching chemistry and medicine. Recognizing that to understand physiologic chemistry students must first be informed upon points not refrred to in most medical text-books, the author has included in his work the latest views of equilibrium of equations, mass-action, cryoscopy, osmotic pressure, dissociation of salts into ions, the effects of ionization upon electric conductivity, and the relationship between purin bodies, uric acid, and urea. Chemical substances he has treated from the standpoint of the medical student and physician, giving much more space to Toxicology than is given in any other text-book on chemistry. The chapters on the clinical chemistry of milk, gastric contents, and the urine, and that on water supply and filtration are full of practical information. Dr. Holland's work will undoubtedly be gladly received by the profession, presenting as it does the mature experience of a practical teacher.

---

Diseases of the Blood (*Anema, Chlorosis, Leukemia, Pseudoleukemia*). By Dr. P. Ehrlich, of Frankfort-on-the-Main; Dr. A. Lazarus, of Charlottenburg; Dr. K. von Noorden, of Frankfort-on-the-Main; and Dr. Felix Pinkus, of Berlin. Entire volume edited, with additions, by Alfred Stengel, M. D., Professor of Clinical Medicine, University of Pennsylvania. Octavo volume of 714 pages, fully illustrated. Philadelphia and London: W. B. Saunders & Co., 1905. Cloth, $5, net; half Morocco, $6, net.

This volume on the Diseases of the Blood is the ninth in Nothnagel's Practice to be published in English. It includes Anemia, Chlorosis, Leukemia, Chloroma, Pseudoleukemia, and each condition is treated so exhaustively and the theories discussed so carefully that the work will remain the last word on the several subjects for many years. Dr. Alfred Stengel, under whose excellent supervision the entire series is being issued, is also the individual editor of this volume. His wide experience and recognized ability as a clinician, and his valuable work concerning the histology, both normal and pathologic, of the blood, renders this volume of unusual interest. His additions are particularly frequent in the article on Anemia. When this esries is completed—and the publishers assure us that the three remaining volumes will shortly appear—it will undoubtedly form the best practice of medicine in existence, expressing the opinions of the highest German and English speaking authorities.

---

A Reference Handbook for Nurses. By Amanda K. Beck, of Chicago. 32mo volume of 150 pages. Philadelphia and London: W. B. Saunders & Co., 1905. Bound in flexible morocco, $1.25, net.

This little book contains information upon every question that comes to a nurse in her daily work, and embraces all the information that she requires to carry out any directions given by the physician; it includes also instructions for all emergencies that may arise before or between visits of the physician. It is of immense value to student nurses because it contains all the material they are expected to commit to memory from notes. Physicians, too, will find the book of value, because it contains exact details as to the solutions, foods, dosage, poultices, applications, etc. There are also articles on bacteriology, massage, medical electricity, obstetrics, care of infants, and such information. The mechanical get-up of the book is both convenient and attractive. It is of a size to fit the pocket and is neatly bound in flexible morocco.

---

Welch & Schamberg on Acute Contagious Diseases.—A Treatise on Acute Contagious Diseases. By William M. Welch, M. D., Consulting Physician to the Municipal Hospital for Contagious and Infectious Diseases; Diagnostician to the Bureau of Health, etc., Philadelphia, and Jay F. Schamberg, A. B., M. D., Profesor of Dermatology and of Infectious Eruptive Diseases, Philadelphia Polyclinic; Consulting Physician to the Municipal Hospital for Contagious and Infectious Diseases, and Assistant Diagnostician to the Philadelphia Bureau of Health, etc. In one very handsome octavo volume of 781 pages, illustrated with 109 engravings and 61 full-page plates. Cloth, $5, net; leather, $6, net; half Morocco, $6.50, net. Lea Brothers & Co., Publishers, Philadelphia and New York, 1905.

The authors, from years of faithful study and abundant clinical experience, are peculiarly well equipped to furnish precisely the practical informtion which the every-day physician needs, and they have succeeded in presenting this knowledge fully and clearly in a style of diction which makes reading a pleasure as well as a profit. The Philadelphia Municipal Hospital offers almost unlimited opportunities for the consideration of Contagious Diseases, and the work is based upon the personal study of the many patients who come daily under the charge of the authorities; thus there have been studied nearly ten thousand cases each of smallpox, scarlet fever and diphtheria in addition to the very many cases of the other diseases discussed, such as vaccinia, measles, chickenpox, rubella, typhus fever, etc.

---

MALFORMATIONS OF THE GENITAL ORGANS OF WOMAN. By Ch. Debierre, Professor of Anatomy in the Medical Faculty at Lille. Eighty-five illustrations. Translated by J. Henry C. Simes, M. D., Emeritus Professor of Genito-Urinary and Venereal Diseases in the Philadelphia Polyclinic. Cloth, 182 pages, $1.50. B. Blakiston's Son & Co., Philadelphia, 1905.

Aside from the curiosity that attaches to a work of this kind, it has a scientific and practical interest of the first order. It will interest you, doctor.

